# Exploring the potential mechanisms of m6A modification in septic acute respiratory distress syndrome: a bioinformatics analysis

**DOI:** 10.1186/s41065-025-00480-x

**Published:** 2025-06-23

**Authors:** Shaoyang Zhang, Qinghui Fu, Zhipeng Xu, Mingjie Fu, Jianfeng Zhao, Wenqiao Yu

**Affiliations:** 1 The Department of Emergency, The First Affiliated Hospital, Zhejiang University School of Medicine, Qingchun Street 79th, Hangzhou, Zhejiang Province 310003 China; 2 The Department of SICU, The First Affiliated Hospital, Zhejiang University School of Medicine, Qingchun Street 79th, Hangzhou, Zhejiang Province 310003 China

## Abstract

**Background:**

Acute respiratory distress syndrome (ARDS) remains a leading cause of mortality in intensive care units. The N6-methyladenosine (m6A) mRNA modification is critical in various pathological conditions, yet its role in the ARDS microenvironment, particularly at the single-cell level, remains poorly understood.

**Methods:**

Single-cell and bulk RNA-sequencing datasets were sourced from the GEO databases. Bioinformatics and experimental approaches were employed to investigate the associations between m6A regulators and hub genes in ARDS.

**Results:**

WTAP, HNRNPA2B1, and HNRNPC exhibited extensive expression within the ARDS microenvironment. Consensus clustering analysis segregated patients with sepsis into distinct subgroups, with WTAP showing significant variation across these groups. Weighted gene co-expression network analysis (WGCNA) identified the brown module as most associated with WTAP, revealing five hub genes. Validation experiments confirmed high expression levels of WTAP and MYC in lung tissues. Functional assays further demonstrated that WTAP enhances ARDS progression.

**Conclusions:**

In conclusion, bioinformatics analysis and preliminary experimental data suggest that WTAP promotes ARDS onset and progression by regulating m6A methylation and facilitating immune cell infiltration.

## Introduction

Acute respiratory distress syndrome (ARDS) is a clinical syndrome marked by respiratory failure, resulting from various etiologies such as pneumonia, sepsis, trauma, and viral infections, and is associated with high morbidity and mortality rates [1]. Sepsis is a key contributor to the development of ARDS, responsible for approximately 32% of ARDS cases in intensive care units [2]. Septic ARDS is typically characterized by excessive immune system activation, leading to pulmonary inflammation, edema, alveolar injury, and often respiratory failure [3]. To date, no specific therapy has been shown to effectively treat sepsis-induced ARDS [4]. Therefore, investigating ARDS resulting from sepsis due to bacterial infection is crucial for advancing both sepsis and ARDS management and understanding.

Single-cell RNA sequencing (scRNA-seq) provides a powerful approach to identifying diverse cell subtypes and discovering novel cell types and states [5]. Previous studies have utilized scRNA-seq to analyze immune cell composition in patients with ARDS, particularly by examining peripheral blood mononuclear cells (PBMCs) [6]. scRNA-seq provides valuable insights into the immune cell populations within the ARDS microenvironment, particularly highlighting endothelial cells, monocytes, macrophages, and T cells. These key immune cells’ behavior is significantly modulated by N6-methyladenosine (m6A) modification, the most prevalent chemical modification in both protein-coding and non-coding RNAs [7]. Accumulating evidence indicates that m6A regulators play a role in ARDS [8]. For example, LPS induces time-dependent increases in m6A methylation in human pulmonary artery endothelial cells, with METTL3 being identified as the primary driver of RNA methylation upregulation [9]. These findings suggest that m6A regulators could serve as potential therapeutic targets for ARDS.

In this study, two datasets, GSE151263 [10] and GSE65682 [11], were acquired from the GEO database. GSE151263, a single-cell sequencing dataset specifically focused on ARDS, was selected for scRNA-seq analysis. GSE65682, a chip dataset related to sepsis, includes detailed survival data and was used to validate the findings. Through comprehensive bioinformatics analysis, this study utilized scRNA-seq data to explore the role of m6A modifications in ARDS. The results from this study may provide valuable insights into the diagnosis and treatment of ARDS, offering a reference for future research and clinical application.

## Materials and methods

### Data acquisition

A single-cell dataset, GSE151263, corresponding to septic ARDS, was retrieved from the GEO database (https://www.ncbi.nlm.nih.gov/geo/) [12], comprising three septic ARDS samples. Additionally, bulk RNA-seq data, GSE65682, focused on sepsis, were also accessed from the GEO database, including 241 sepsis samples resulting from abdominal and lung infections. Clinical and survival data were also collected.

### Single cell sequencing processing

Using the Seurat package [13], this study constructed the object and removed low-quality cells. Standard data preprocessing was performed to compute the percentage of gene numbers, cell counts, and mitochondrial sequencing counts. Genes detected in fewer than three cells were excluded, as were genes present in fewer than 200 cells. Cell clustering was performed using the “FindClusters” function from the Seurat R package.

### Score according to phenotype-related hallmark genes

To assess module scores and the enrichment fraction for phenotype-related gene expression in single cells, AUCell was applied [14]. Hallmark genes related to necroptosis, ferroptosis, apoptosis, pyroptosis, methylation, inflammation, angiogenesis, autophagy, and hypoxia were retrieved from the GeneCard database (https://www.genecards.org/) [15], which provides extensive data on gene functions. Genes with a correlation greater than 10.0 were selected for bioinformatics analysis.

### Cell trajectory and cell communication analysis

To elucidate the mechanisms underlying functional alterations and explore potential lineage divergence across distinct clusters, Monocle2 was used for pseudotemporal analysis of cellular progression [16]. The pseudotime was computed using the Monocle2 algorithm and normalized to a range from 0 to 1. The “differentialGeneTest” was applied to identify hub genes within each cluster. To investigate cell communication interactions and the mechanisms behind communication molecules at the individual cell resolution, the “italk” R package was used for cell-cell communication correlation analysis [17].

### Consensus cluster analysis to build clusters based on the differential genes by cell trajectory and Immune cell infiltration

Consensus clustering analysis was conducted on the GSE65682 cohort based on differential expression genes from cell trajectory analysis, utilizing the “ConsensusClusterPlus” R package. The results showed that partitioning the samples into three clusters (k = 3) provided the most optimal intra-cluster associations, minimal coefficient of variation, and adequate sample sizes within each cluster. The stromal and immune cell estimations were performed using the ESTIMATE algorithm. Stromal, immune, and ESTIMATE scores for sepsis were calculated using the “estimate” R package. To examine the correlation between immune cell infiltration and consensus clusters, the CIBERSORT algorithm (http://www.cibersort.stanford.edu/) was employed to assess immune cell infiltration in patients with GEO-sepsis. The Wilcoxon test was used to analyze differences in immune cell infiltration among the three groups. Similarly, the infiltration discrepancies of specific functional cells in different score groups were evaluated using the same method.

### WGCNA

Gene coexpression networks for the GSE65682 dataset were constructed using the R package “WGCNA” in conjunction with the gene expression profiles [18]. The network construction process involved several key steps: (1) Defining the similarity matrix. (2) Selecting a weight coefficient β = 12 to convert the similarity matrix into an adjacency matrix. (3) Transforming the adjacency matrix into a topological overlap matrix (TOM). (4) Layering the dissTOM based on Tom Cluster to obtain a hierarchical clustering tree. (5) Applying a dynamic tree-cut method to identify modules from the hierarchical clustering tree. (6) Calculating module eigengenes (MEs) for each module, representing the overall expression level of the module.

### Preliminary experimental verification

Thirty adult male BALB/c mice, aged 8 weeks, were provided by the animal experimental center of Zhejiang University (Hangzhou, China). All animal study protocols were approved by the animal care and use committee of Zhejiang University, and all methods were performed in accordance with relevant guidelines and regulations, as well as the ARRIVE guidelines. The mice underwent intratracheal instillation of 2.4 mg/kg of LPS in saline (or saline as control) under anesthesia with sodium pentobarbital (40 mg/kg). After 12 h of LPS stimulation, the mice were euthanized under anesthesia. The right upper lobe lung tissues of each group were harvested, and surface water was removed using filter paper. The wet-to-dry weight ratio (W/D) of lung tissue was calculated to assess the extent of pulmonary edema. The levels of TNF-α, IL-6, IL-1β, and NO in the bronchoalveolar lavage fluid (BALF) of mice were measured using ELISA kits according to the manufacturer’s instructions. Total RNA was extracted from the cells using TRIzol reagent (Invitrogen). Reverse transcription of mRNA into cDNA was carried out using a reverse transcription reagent, following the commercial kit guidelines. m6A RNA methylation quantification was performed using an m6A RNA methylation quantification kit (Abcam) as per the manufacturer’s instructions.

### Statistical analysis

Continuous variables were compared using Student’s t-test for normally distributed data or the Mann-Whitney U test for non-normally distributed data. Spearman’s correlation coefficient was used to assess correlations between continuous variables. Statistical analyses were conducted using SPSS 19.0 software (IBM, Armonk, NY, USA). A P-value < 0.05 was considered statistically significant.

## Result

### Annotation of 11 types of cells in ARDS

**Fig. 1:**
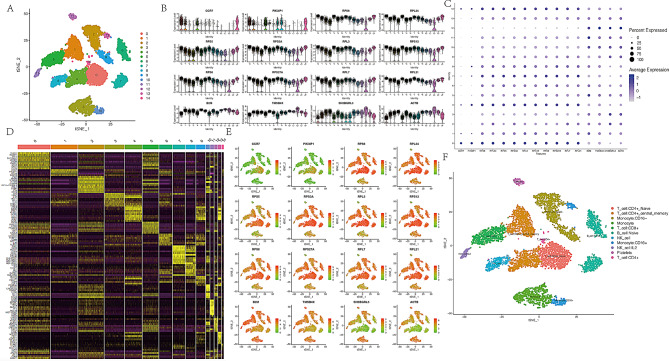
Overview of dimensionality reduction cluster analysis and feature description of single-cell sequencing data. (**A**) Identification of 14 cell clusters via dimensionality reduction cluster analysis. (**B**) and (**C**) Expression of the top marker genes in each cell cluster. (D) Heatmap illustrating the top 10 differential genes in each cell type. (**E**) t-SNE map displaying the expression distribution of top marker genes. (**F**) t-SNE map depicting the distribution of 11 identified cell types. tSNE: t-distributed stochastic neighbor embedding

A comprehensive screening of 8,056 cells and 16,432 genes was performed using the GSE151263 dataset. Cell clustering and the identification of 15 clusters were achieved using the FindCluster function (Fig. 1A). The expression levels of the top 16 most significantly upregulated marker genes in each cluster are visually represented in Figs. 1B, C, and E, while the top 10 prominent marker genes within each subgroup are displayed in Fig. 1D. These 15 clusters were then annotated into 11 distinct cell types: CD4 + Naïve T cells, CD4 + central memory T cells, Monocyte CD16-, Monocytes, CD8 + T cells, Naïve B cells, NK cells, IL2 NK cells, Platelets, CD4 + T cells, and CD16 + Monocytes. These cell types were identified and annotated based on characteristic markers, as shown in Fig. 1F.

### The score of each phenotype and m6a-related genes expression

**Fig. 2:**
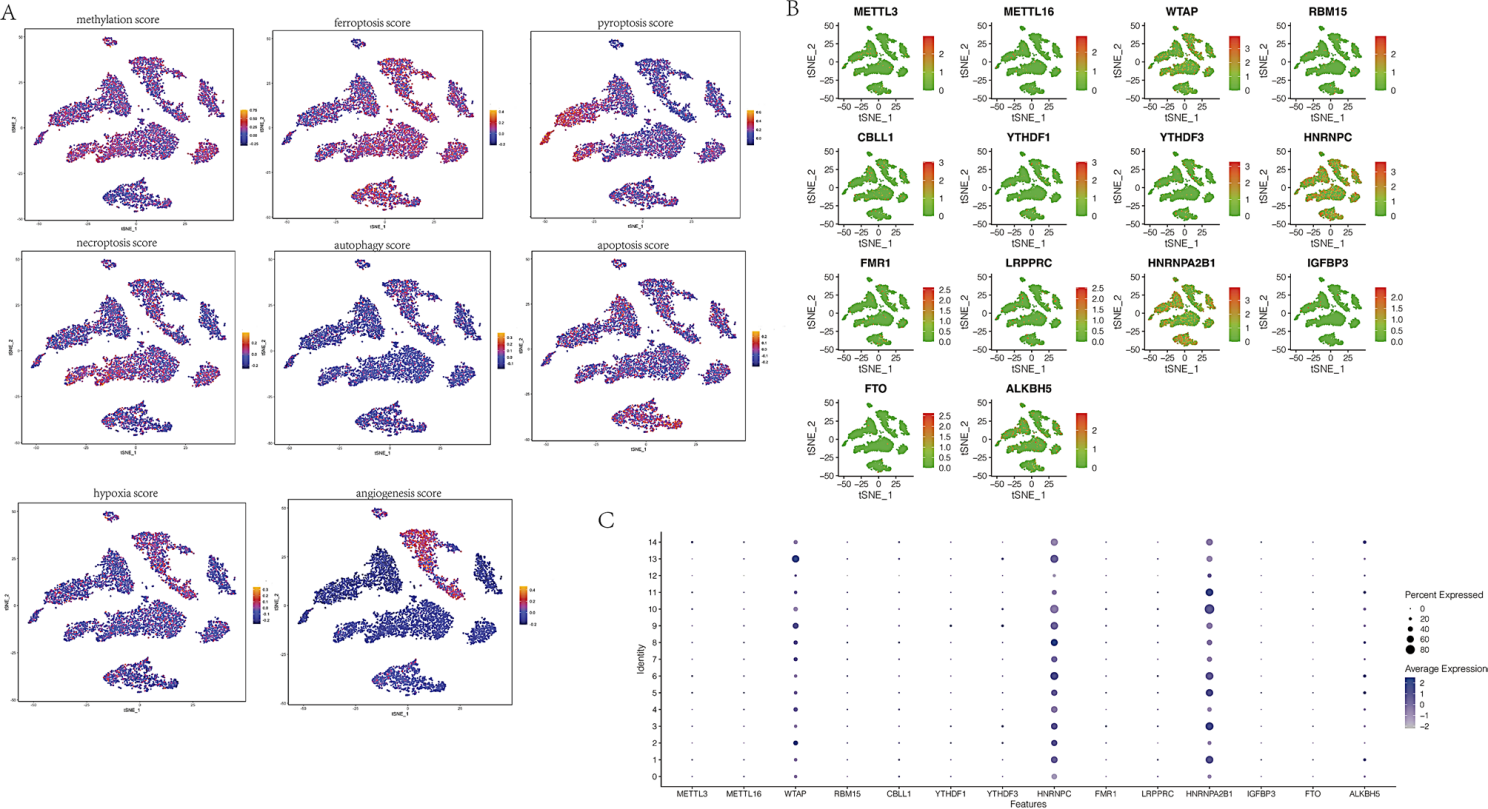
(**A**) Pyroptosis score, ferroptosis score, necroptosis score, autophagy score, apoptosis score, m6A methylation score, hypoxia score, and angiogenesis score calculated using the AUCell function. The lighter the color, the higher the score; conversely, the darker the color, the lower the score. (**B**) Distribution of m6A regulators in the ARDS microenvironment. Green indicates low expression and orange indicates high expression. (**C**) Expression levels of m6A regulators across the 11 cell types. The darker the color, the higher the expression; the lighter the color, the lower the expression

Scores for pyroptosis, ferroptosis, necroptosis, autophagy, apoptosis, m6A methylation, hypoxia, and angiogenesis were calculated. Pyroptosis-related scores were significantly higher in NK and CD8 + T cells compared to other cell types, as determined by the AUCell function (Fig. 2A). Ferroptosis and m6A methylation were observed across all cell types (Fig. 2A). Necroptosis-related scores were notably elevated in CD4 + Naïve T cells and NK cells (Fig. 2A). Autophagy was absent in all cell types (Fig. 2A). Apoptosis-related scores were significantly higher in Monocytes and CD16 + Monocytes (Fig. 2A), while hypoxia and angiogenesis-related scores were elevated in Monocyte CD16- (Fig. 2A).

Given that m6A methylation had the highest score, further analysis was performed. Expression levels of m6A regulators across all cell types in septic ARDS were examined, and t-SNE plots of these m6A regulators are shown in Fig. 2B. Notably, WTAP, HNRNPA2B1, and HNRNPC exhibited higher expression across all cell types, whereas other m6A regulators showed minimal or no significant expression (Fig. 2C).

### Cells communications analysis and cell trajectory of microenvironment

**Fig. 3:**
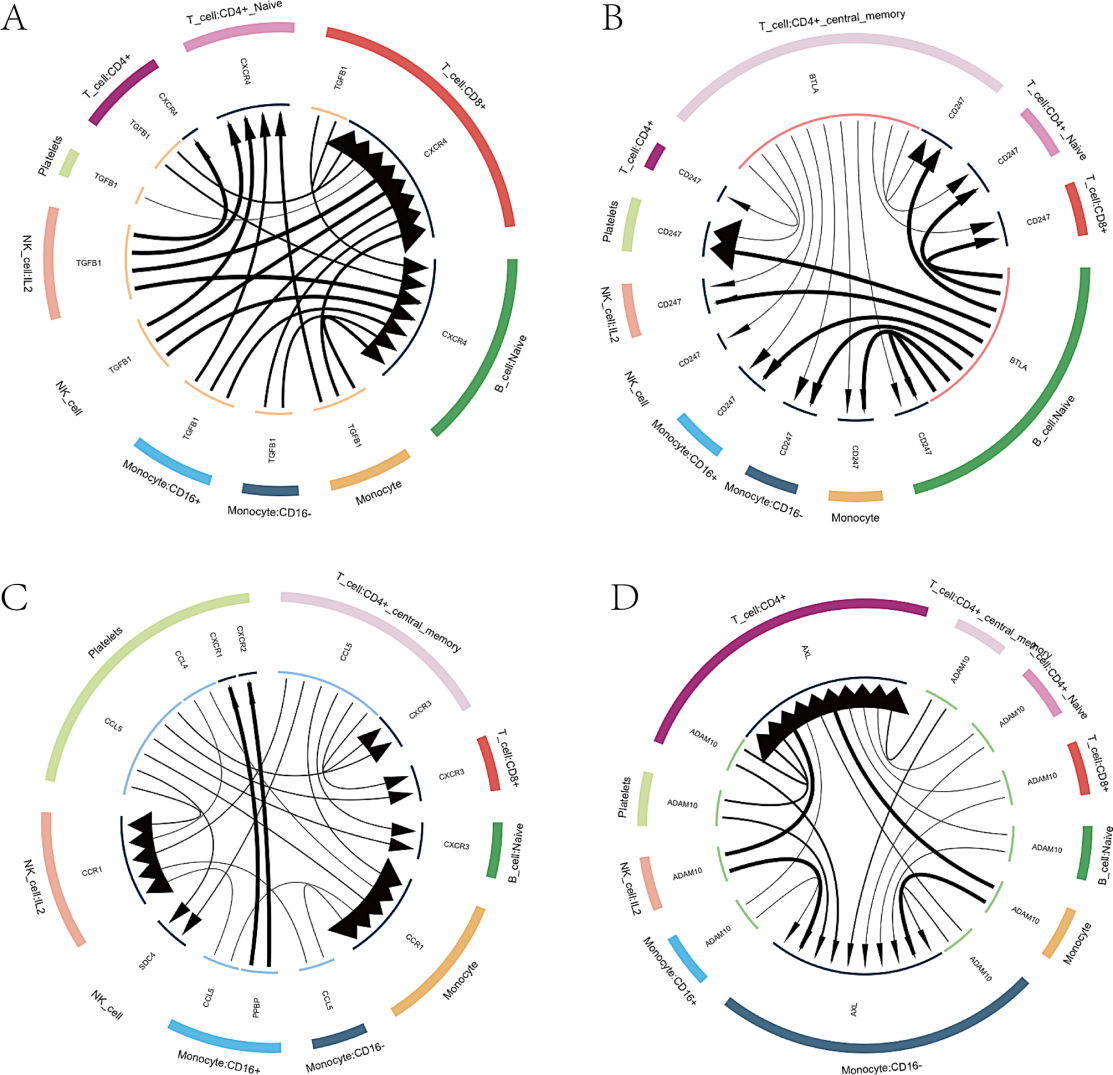
(**A**) Intracellular and intercellular communications in the growth factor module of cell communication networks. (**B**) Immune checkpoint module of cell communication networks. (**C**) Cytokine module of cell communication networks. (**D**) Other module of cell communication networks. Node (square): Represents different cell types. Connecting lines (lines): They represent the communication relationship between cells, and their thickness directly reflects the intensity of communication (thick lines = strong communication, thin lines = weak communication)

To elucidate the molecular associations between cells, cell communication networks were initially established using iTALK to analyze interactions of potential receptor-ligand pairs. Cellular interactions were then assessed within four distinct modules: “Growth Factor” (Fig. 3A), “Immune Checkpoint” (Fig. 3B), “Cytokine” (Fig. 3C), and “Other” (Fig. 3D), based on the specific functions of the receptor-ligand pairs. In the Growth Factor module, significant intercellular communication was observed between CD8 + T cells and Naïve B cells, suggesting the potential influence of autocrine or paracrine growth factors on these cells. Notably, both CD8 + T cells and Naïve B cells exhibited higher levels of CXCR4 expression compared to other cell types. This indicates that these cells may contribute to the progression of ARDS through activation of the TGFB1-CXCR4 signaling pathway. In the Immune Checkpoint module, platelets displayed considerable intercellular communication and higher CD247 expression relative to other groups. Platelets may play a role in ARDS progression by activating the BTLA-CD247 signaling pathway. In the Cytokine module, CCR1 expression was notably higher in monocytes and IL2 NK cells than in other cell types. In the “Other” module, AXL was expressed at higher levels in CD4 + T cells compared to other cell types.

**Fig. 4:**
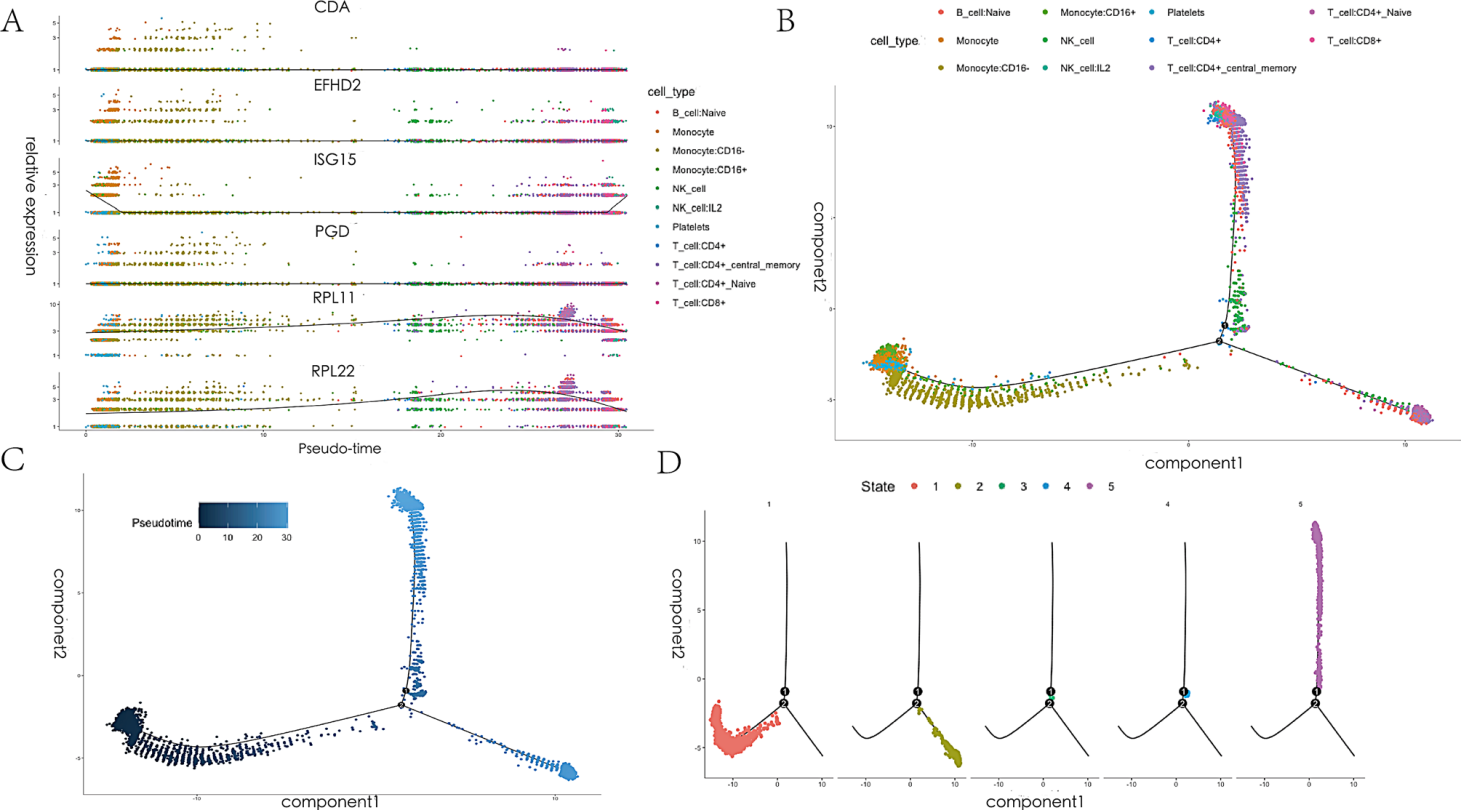
(**A**) Scatter plot showing the dynamic expression of the top 6 genes across different cells with pseudotime values. (**B**) Distribution of various cell types in the trajectory curve. (**C**) Cell cluster distribution in the principal component analysis. The depth of color indicates the ordering of cells according to their pseudotime values. (**D**) Distribution of cells in different states. Monocle divides the trajectory curve into distinct states and analyzes differential gene expression in each state

Cell trajectories associated with the immune microenvironment of ARDS were analyzed to gain further insight into cellular progression. Figure 3 C illustrates the distribution of cell clusters in the principal component, with color depth indicating the ordering of cells according to their pseudotime values. Figure 3B shows the distribution of different cell types along the trajectory curve, with monocytes and NK cells being more prevalent initially, followed by a predominance of CD4 + and CD8 + T cells over time. Figure 3D displays the distribution of cells in different states, with Monocle dividing the trajectory curve into distinct states and analyzing the differential gene expression of cells in each state. Figure 4 A presents a scatter plot showing the dynamic expression of the top six genes in various cells across pseudotime values.

### Enrichment analyses of differentially expressed genes and consensus clustering and immune microenvironment landscape analysis

**Fig. 5:**
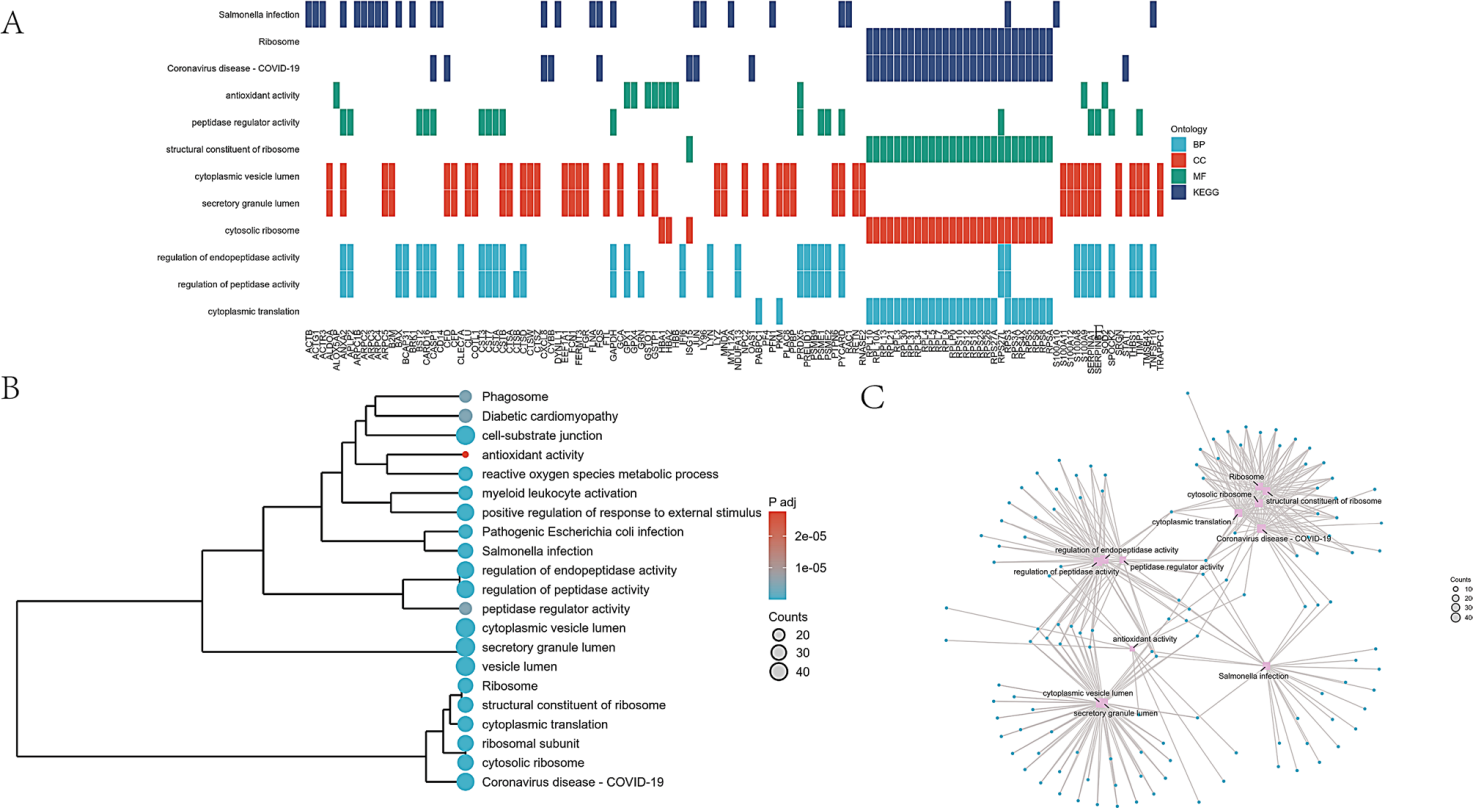
(**A**), (**B**), and (**C**) Top 3 significant differences in biological processes (BP), cellular components (CC), molecular functions (MF), and pathways. KEGG: Kyoto Encyclopedia of Genes and Genomes

Gene Ontology (GO) analysis revealed that the top three biological processes (BPs) were the regulation of endopeptidase activity, regulation of peptidase activity, and cytoplasmic translation. The most prominent cellular component (CC) terms were cytoplasmic vesicle lumen, secretory granule lumen, and cytosolic ribosome. The leading molecular function (MF) term identified was the regulation of antioxidant activity. In the KEGG pathway analysis, the top three pathways associated with the differential genes were Salmonella infection, ribosome function, and coronavirus disease-19 (Fig. 5A, B, and C).

**Fig. 6:**
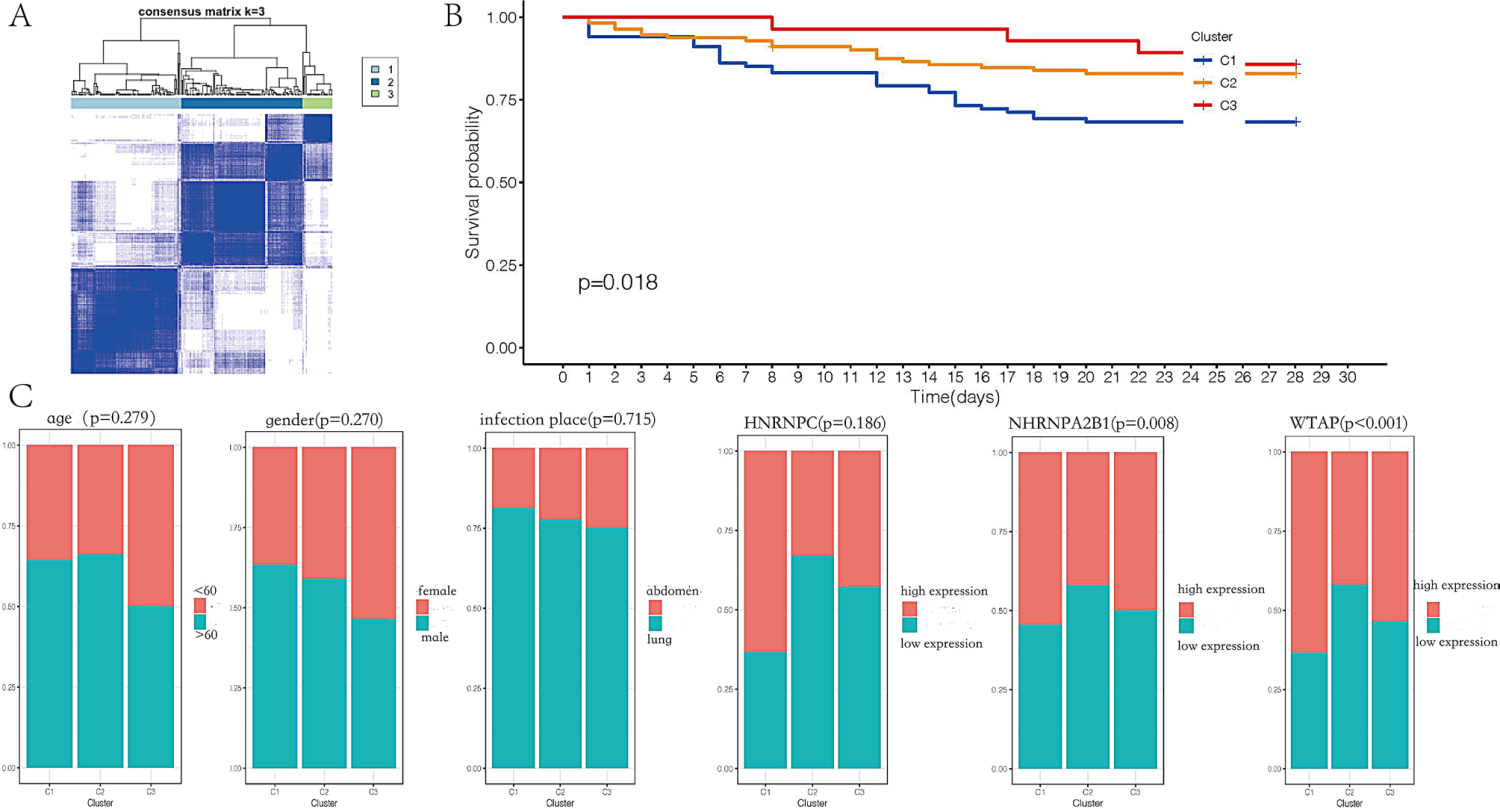
(**A**) Consensus clustering analysis was used to categorize patients with sepsis into three subgroups. (**B**) Kaplan-Meier survival curve comparing the three clusters of patients with sepsis. (**C**) Distribution of different parameters across the three subgroups

**Fig. 7:**
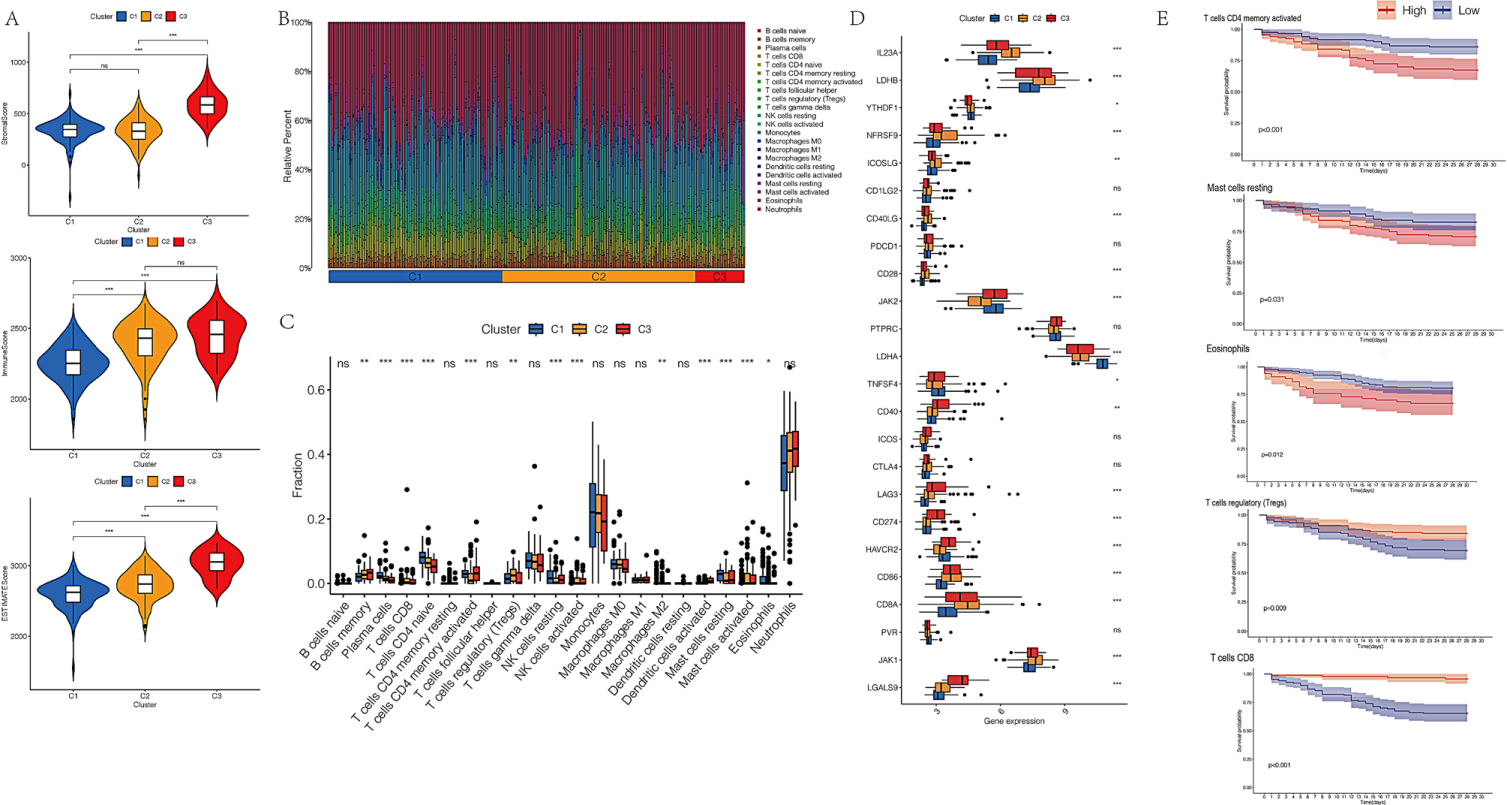
(**A**) Violin plot showing stromal score, immune score, and ESTIMATE score across the three groups. (**B**) and (**C**) Proportions of 22 immune cell types in the three groups, determined by the CIBERSORT method. (**D**) Immune checkpoint analysis between the three groups. (**E**) Kaplan-Meier survival curves showing significant differences in the top 5 cell types

ARDS, a severe complication of sepsis, is associated with a poor prognosis. To assess the prognostic value of differential genes identified through immunomicroenvironment analysis of ARDS induced by sepsis, confirmatory analyses were performed using the GSE65682 dataset. Due to the absence of an ARDS dataset with survival prognosis data in the GEO database, the sepsis dataset GSE65682 was employed for this analysis. Given the heterogeneity of the immune microenvironment in sepsis, identifying high-risk patients is crucial. Consensus clustering analysis was used to categorize patients with sepsis into subgroups based on the differential gene expression, providing insights into the role of these genes in ARDS induced by sepsis. The analysis revealed that partitioning patients into three subgroups (K = 3) yielded the most optimal results (Fig. 6A). The Kaplan-Meier survival curve showed that patients in Cluster 1 had a lower overall survival (OS) rate compared to those in Cluster 2 and Cluster 3 (Fig. 6B). No significant differences were observed in age, gender, infection site, or expression levels of HNRNPC across the three clusters. However, significant differences in the expression levels of WTAP and HNRNPA2B1 were detected between the clusters (Fig. 6C). The ESTIMATE algorithm was applied to explore the immune microenvironment across the three clusters. Patients with sepsis in Cluster 1 had lower stromal, immune, and ESTIMATE scores compared to patients in the other two clusters (Fig. 7A).

CIBERSORT analysis was performed to evaluate the immune infiltration landscape in the three clusters. As shown in Figs. 7B and C, the CIBERSORT algorithm revealed that Cluster 1 had higher proportions of plasma cells, CD4 + Naïve T cells, resting NK cells, M2 macrophages, and eosinophils. In contrast, Cluster 3 exhibited higher proportions of memory B cells, activated CD4 + T cells, and dendritic cells. A total of 24 immune checkpoint genes were assessed in GSE65682. Cluster 1 exhibited significantly lower expression levels of IL23A, NFRSF9, ICOSLG, CD40LG, CD28, LAG3, CD274, CD86, CD8A, JAK1, and LGALS9, while exhibiting a significantly higher expression of LDHA (Fig. 7D). Furthermore, an increase in activated CD4 + T cells and CD8 + T cells was positively correlated with improved prognosis, while higher levels of regulatory T cells, resting mast cells, and eosinophils were associated with worse prognosis (Fig. 7E).

### Identification of hub-genes

**Fig. 8:**
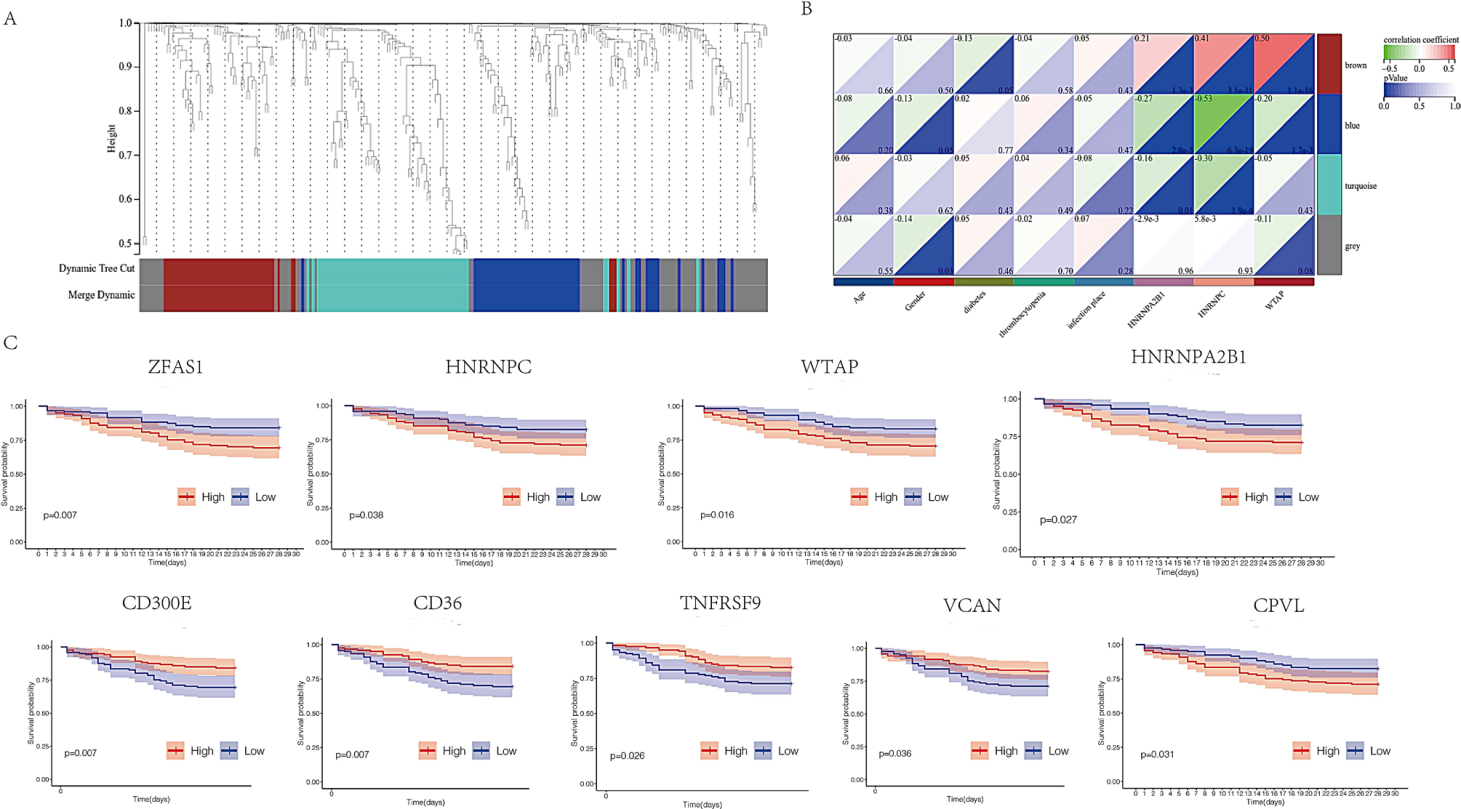
(**A**) Four modules were identified through Weighted Gene Co-expression Network Analysis (WGCNA). (**B**) The brown module showed the strongest association with WTAP expression. (**C**) Kaplan-Meier survival curves for WTAP, MYC, CPVL, HNRNPA2B1, HNRNPC, CD300E, CD36, TNFRSF9, and VCAN

To further explore the relationship between differential genes and m6A methylation, WGCNA analysis was performed. Gene coexpression networks for patients with sepsis were constructed using the R package “WGCNA.” Cluster analysis was then conducted on the modules, and modules with similar distances were merged into a new module (parameters set to height = 0.25, deepSplit = 3, minModuleSize = 100), resulting in a total of four modules. The grey module, identified in the cluster analysis, represented a gene set that could not be merged with other modules (Fig. 8A). Correlation analysis between each module revealed that the brown module exhibited the strongest association with WTAP expression (Fig. 8B). The top six genes in the brown module were CD300E, TNFRSF9, VCAN, MYC, CD36, and CPVL. The Kaplan-Meier survival curve showed that patients with higher expression levels of WTAP, MYC, CPVL, HNRNPA2B1, and HNRNPC had a lower OS rate compared to those with lower expression levels. Conversely, patients with higher expression levels of CD300E, CD36, TNFRSF9, and VCAN had a higher OS rate than those with lower expression levels (Fig. 8C).

### Preliminary experimental verification

**Fig. 9:**
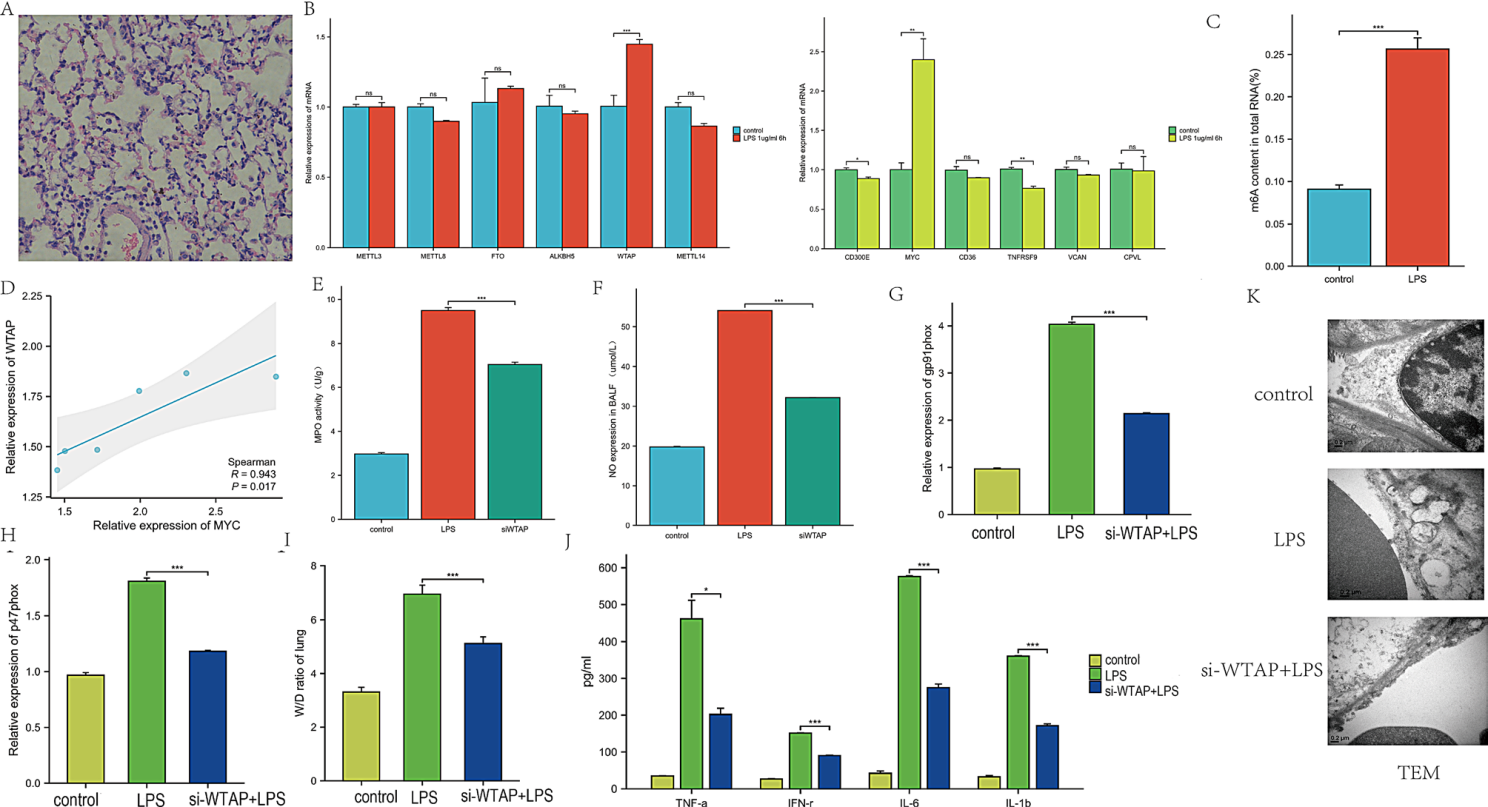
Preliminary experimental validation. (**A**) Lung tissue samples from mice after intratracheal instillation of 2.4 mg/kg LPS. (**B**) mRNA expression levels of m6A regulators and hub genes in lung tissues. (**C**) Correlation analysis showing a positive correlation between MYC and WTAP expression. (**D**) ELISA measurement of mRNA m6A levels in control and LPS-treated groups. (**E**) Myeloperoxidase (MPO) activity in different groups. (**F**) Nitric oxide (NO) expression in different groups. (**G**) and (**H**) Expression of gp91phox mRNA and p47phox mRNA in different groups. (**I**) Wet-to-dry ratio observed between the three groups. (**J**) Inflammatory cytokines in BALF, measured by ELISA. (**K**) Glycocalyx structure of lung capillaries in different groups

To clarify the functional significance of m6A modification in sepsis-induced ARDS, an assessment was conducted in mouse lung tissues (Fig. 9A). The results showed a significant increase in m6A levels in total RNA from septic lungs, as determined by colorimetric ELISA (Fig. 9D). Since m6A modification is primarily regulated by m6A writers and erasers, it was hypothesized that the dysregulation of these genes might contribute to the observed elevation of m6A RNA levels in septic lungs. Notably, the methyltransferase WTAP was significantly upregulated in septic lungs (Fig. 9B). Further analysis of the mRNA expression levels of hub genes in lung tissues revealed that MYC was significantly upregulated in septic lungs. A positive correlation was observed between the expression levels of MYC and WTAP (Fig. 9C).

To investigate the role of WTAP in sepsis-induced ARDS, WTAP-knockdown models were generated in mice by intranasally administering WTAP siRNA or negative control (NC) siRNA. The knockdown of WTAP resulted in a significant reduction in myeloperoxidase (MPO) activity and nitric oxide (NO) expression in septic mice (Fig. 9E and F). In comparison to the control group, the expression levels of NOX subunits, specifically gp91phox mRNA and p47phox mRNA, were elevated in the siNC + LPS group. In contrast, the si-WTAP group showed reduced expression of gp91phox mRNA and p47phox mRNA compared to the siNC + LPS group (Fig. 9G and H). Additionally, a marked difference in the wet-to-dry ratio was observed between the siNC + LPS group and the si-WTAP group (Fig. 9I). The levels of inflammatory cytokines in BALF significantly decreased following WTAP siRNA administration, compared to NC siRNA administration (Fig. 9J). Furthermore, transmission electron microscopy (TEM) analysis revealed varying degrees of damage to the glycocalyx structure of lung capillaries, with a significant increase in glycocalyx layer thickness observed in the si-WTAP group compared to the siNC + LPS group (Fig. 9K). Collectively, these results suggest that the hypermethylation of WTAP exacerbates endothelial barrier dysfunction and amplifies the inflammatory response induced by sepsis.

## Discussion

The primary pathological feature in the development of acute lung injury (ALI) is the onset of an inflammatory storm [19]. Managing ARDS, especially through immunotherapy, remains a significant challenge [20]. While the involvement of m6A modification in ARDS has been established, the application of novel techniques such as single-cell analysis is essential to uncover the potential mechanisms of m6A modification within the ARDS microenvironment [21]. However, there is a lack of comprehensive studies that characterize the m6A modification landscape in the ARDS microenvironment at the single-cell level. In this study, single-cell RNA-seq datasets were analyzed to identify 11 distinct cell types within the ARDS microenvironment. The relationships between m6A regulators, functional states, and cell communication within these cell types were also examined. Additionally, cell trajectory analysis was employed to identify differentially expressed genes in the ARDS microenvironment. By utilizing bulk RNA-seq datasets, three distinct patient clusters with differential gene expression were identified. To further explore whether these differential genes are related to m6A methylation, WGCNA analysis was performed. Our preliminary findings suggest that WTAP-related m6A modification contributes to lung injury and endothelial barrier dysfunction. Moreover, our analysis indicates that WTAP may influence MYC hypermethylation, mediating the progression of ARDS.

In this study, patients in Cluster 1 from the GSE65682 dataset exhibited lower OS rates, as well as reduced stromal and immune scores. According to current theories, the pathogenesis of ARDS involves the degradation of alveolar endothelial and epithelial tissue, facilitated by platelet-derived products and innate immune cells [22]. This may explain the observed lower stromal score and poorer OS rate. However, the exact role of adaptive immune cells in ARDS progression remains unclear. CD8 + T cells, which are cytotoxic in nature, can secrete cytokines and perforin to eliminate virus-infected cells [23]. Our findings revealed a significant reduction in CD8 + T cell numbers and immune scores in non-survivors compared to survivors, suggesting a diminished protective effect. Lei et al. have previously reported that CD8 + T cells are promising independent prognostic markers for sepsis-induced ARDS, with a heightened state of CD8 + T cell exhaustion strongly correlating with poor prognosis [24]. Regulatory T cells (Tregs), typically found in lymphoid tissues and peripheral blood, have been shown to play a critical role in modulating the inflammatory response. Leukotrienes B4 (LTB4) have the ability to attract CD4 + CD25 + Foxp3 + Tregs, thereby reducing inflammation associated with ALI [25, 26]. In general, Tregs communicate with other T cells through the secretion of anti-inflammatory factors, such as IL-10, which helps protect the lungs from injury during transfusion [27]. Furthermore, Tregs can inhibit the proliferation of ALI-related fibers by limiting the recruitment of fibrocytes [28]. CD39 + Tregs alleviate lipopolysaccharide (LPS)-induced ALI through autophagy and ERK/FOS signaling pathways [29]. Single-cell analysis revealed significant levels of ferroptosis and hypermethylation across most cell types, suggesting a potential link between T cell ferroptosis induced by hypermethylation and the progression of ARDS. Scholarly discussion on the distribution of m6A regulators across various cell types and throughout different cell cycle phases remains limited. Analysis of the GSE151263 dataset revealed that most m6A regulators exhibit elevated expression levels in ARDS, particularly HNRNPA2B1, HNRNPC, and WTAP. Additionally, the GSE65682 dataset demonstrated significant differences in the expression levels of WTAP and HNRNP2B1 among the three patient groups, suggesting a potential link between m6A modification and ARDS progression. Previous research has established an association between m6A methylation and the pathogenesis of lung ischemia-reperfusion injury [30]. Another study highlighted the connection between m6A modification and the activation of inflammatory pathways, as well as endothelial barrier dysfunction [31]. Feng et al. observed that LPS administration led to a time-dependent increase in m6A methylation in human pulmonary artery endothelial cells, with METTL3 identified as the main contributor to RNA methylation upregulation [9]. In the present study, inhibiting WTAP expression alleviated lung damage in mice. However, the specific cells responsible for ARDS progression remain unidentified, and further research is required. The potential role of T cell hypermethylation in this process also warrants additional investigation.

Notably, MYC was the only gene highly expressed in the lung tissue of ARDS mice. The m6A methylation of MYC has been implicated in various diseases [32–34]. Several studies have established a correlation between WTAP and the m6A modification of MYC. Naren et al. demonstrated that WTAP exerts an epigenetic influence on acute myeloid leukemia cells by specifically regulating the m6A methylation of MYC mRNA [35]. Cao et al. later confirmed that gemcitabine disrupts WTAP protein expression in pancreatic cancer, resulting in decreased m6A modification of MYC, reduced RNA stability, and impeded cancer progression [36]. Tao et al. identified WTAP as a potential prognostic biomarker and suggested its involvement in regulating immune cell infiltration in nasopharyngeal carcinoma [37]. However, the role of WTAP in critical care medicine remains understudied. Qian et al. observed that WTAP may contribute to the acceleration of sepsis development by regulating m6A methylation and promoting immune cell infiltration [38]. These findings imply that m6A modification could be a key player in the regulatory mechanisms of ARDS. However, the exact molecular pathways linking m6A modification to ARDS remain unclear, and further research is necessary to explore whether targeting MYC via m6A regulation could improve ARDS prognosis.

Several limitations should be acknowledged in this study. Firstly, the analyses were based on published datasets, and further experimental validation is needed to confirm the mechanisms underlying m6A modification in the ARDS microenvironment. Secondly, the absence of a publicly available dataset focused on the prognostic data of septic ARDS restricted our analysis to sepsis data, which may limit the study’s persuasive power. Thirdly, sample size of scRNA-seq data (*n* = 3) may reduce the statistical power to detect subtle transcriptional changes in the ARDS microenvironment. While we utilized rigorous bioinformatic methods to mitigate batch effects, the small cohort size increases the risk of overlooking rare cell subpopulations and limits the generalizability of cell-type-specific findings. Future validation in larger cohorts is essential to confirm these preliminary observations.

## Conclusion

In conclusion, WTAP was identified through bioinformatics analysis and preliminary experiments as a key factor that promotes the onset and progression of ARDS by modulating m6A methylation and facilitating immune cell infiltration. These findings provide potential drug targets for the early identification, diagnosis, and treatment of ARDS.

## Data Availability

No datasets were generated or analysed during the current study.
